# High Performance Anti-Corrosion Coatings of Poly (Vinyl Butyral) Composites with Poly *N*-(vinyl)pyrrole and Carbon Black Nanoparticles

**DOI:** 10.3390/ma11112307

**Published:** 2018-11-17

**Authors:** Lu Hao, Guowei Lv, Yaqian Zhou, Kaiming Zhu, Mochen Dong, Yuhang Liu, Demei Yu

**Affiliations:** 1 Department of Applied Chemistry, School of Science, MOE Key Laboratory for Non-Equilibrium Synthesis and Modulation of Condensed Matter, Xi’an Jiaotong University, Xi’an 710049, China; haoluatssd@163.com (L.H.); guoweilv@xjtu.edu.cn (G.L.); zhoupolymer@163.com (Y.Z.); zkm1994@stu.xjtu.edu.cn (K.Z.); mcdong@stu.xjtu.edu.cn (M.D.); 2 State Key Laboratory of Electrical Insulation and Power Equipments, Xi’an Jiaotong University, Xi’an 710049, China; 3 Department of Chemistry, Hong Kong University of Science and Technology, Hong Kong 999077, China; yliuay@outlook.com

**Keywords:** carbon black, poly *N*-(vinyl)pyrrole nanoparticles, corrosion resistant

## Abstract

Zinc is widely used in battery negative electrodes and steel coatings for automotive industries. The anti-corrosion property of zinc is the most important factor determining the performance and lifetime of the products. In this paper, both size-controlled poly *N*-(vinyl)pyrrole (PNVPY) nanoparticles and carbon black (CB) nanoparticles were compounded with poly (vinyl butyral) (PVB) binder developing a series of composite coatings covered on the zinc substrates using a spin-coating technique. The morphologies of the surface and cross section of the PNVPY/CB/PVB coatings indicate that the PNVPY and CB nanoparticles are uniformly distributed in the matrix. The corrosion resistance of the composite coatings was tested by electrochemical impedance spectroscopy (EIS) and potentiodynamic polarization in a 3.5% NaCl solution. It is found that the coating with 1.9 wt.% PNVPY and 2.3 wt.% CB nanoparticles shows a remarkably high resistance value (R_c_) and corrosion protection efficiency (99.99%). Meanwhile, the immersion results also reveal its superior corrosion resistance. It is considered that the nanoscale dispersion of PNVPY and carbon in PVB matrix and the strong interface action between the nanoparticles and PVB result in the uniform microstructure of the composites which endues the superior corrosion properties of the coatings.

## 1. Introduction

Metals corrosion is considered a serious problem in modern civilization [1], especially in the field of the metallurgical and electronic industries. As one of the most popular metals, zinc is widely used in battery negative electrodes and steel coatings for automotive industries. The anti-corrosion property of zinc is the most important factor determining the performance and lifetime of the products. Several strategies have been developed to prepare zinc protective layers. Aramaki’s group prepared a protective film by immersing a zinc electrode in a Ce(NO_3_)_3_ aqueous solution and detected in an aerated 0.5 M NaCl solution by polarization measurement. The protective efficiency of the film against zinc corrosion was more than 91% [2]. Magalhães’s group characterized the morphology and electrochemical features of zinc surfaces converted in acid baths of sodium molybdate by immersion. Corrosion resistance was tested under different conditions. The conversion coatings with 0.3 M molybdate baths at pH 3 acidified with phosphoric acid for 10 min provided the best performance [3]. Although these methods can effectively prevent zinc from corrosion, a little pitting corrosion also occurred on the surface of coating and the performance sometimes was lower [4,5].

Conducting polymers (CPs) are one of the most promising organic corrosion inhibitors as anti-corrosive coating materials owing to their excellent anticorrosion ability that acts as both physical and electronic barriers [6,7,8]. Furthermore, defect-free coatings with uniform coverage may be obtained when the coatings are doped with CPs. DeBerry [9] firstly reported that conductive polymer polyaniline (PANI) promoted the formation of a passive surface on stainless steel and then made stainless steel anti-corrosive in sulfuric acid solution in the 1980s. Syed et al. [10] fabricated polyaniline-polyacrylic acid/polyethyleneimine (PANI-PAA/PEI) composite coatings with a multilayer structure for corrosion protection of 316 stainless steels (316SS). It was found that the PANI-PAA/PEI coating with an optimized layer number of 20 showed improved corrosion protection.

Among the group of conducting polymers, such as polyaniline, polythiophene and polypyrrole, polypyrrole (PPy) and its derivatives are widely studied due to their environmental stability, relatively high conductivity and ease of synthesis by chemical and electrochemical methods [11,12,13]. Ryu et al. [14] prepared a dense polypyrrole film on 55% Al–Zn-coated steel in an acidic tartrate solution by the electrochemical method. The PPy layer can maintain passivation of steel in a 3.5 wt.% NaCl aqueous solution and protect the steel for several hours. Ruhi et al. [15] synthesized polypyrrole/SiO_2_ composite by chemical oxidative polymerization of pyrrole using FeCl_3_ as an oxidant. The results of electrochemistry analysis exhibited a remarkably high corrosion protection efficiency of epoxy coatings with polymer composite in 3.5 wt.% NaCl solution.

Although there are positive reports on the application of PPy in corrosion protection, PPy still has some limitations when used in anti-corrosion coatings. Firstly, PPy has poor solubility in many common solvents due to its rigid molecule structure. As a result, it is difficult to disperse PPy well into the matrix, causing poor antiseptic performance. Secondly, there are always metal ion integrities in the preparation of PPy by conventional methods, resulting in the limitation for its application, especially for corrosion protection. Thirdly, PPy fails to protect coatings when larger defects exist [16]. Usually, preparing composite coatings by adding PPy is in favor of the formation of a passive metal surface at the coating/metal interface [16,17,18]. However, an insulating layer will be formed at the PPy and metal interface when PPy is applied on non-noble metal surfaces, resulting in a low potential caused by electronic decoupling at the interface, which is unfavorable for protracted protection of metal [19,20]. To avoid such problem, CB can be employed as conductive spacers to ensure the electronic contact at the CPs/metal interface [21].

In our previous study, a serious of well-dispersed sulfate doped PPy and its derivative nanoparticles were synthesized by a green method, providing a chance to enlarge their application [22,23]. In this work, both PNVPY and carbon black nanoparticles are filled in poly (vinyl butyral) (PVB) matrix with different contents forming a series of composite coatings. The coatings are then spun on the zinc substrate as a corrosion protection. The electrochemical properties of bare and coated zinc are investigated by open-circuit voltage (OCP), electrochemical impedance spectroscopy (EIS) and potentiodynamic polarization. Meanwhile, the anticorrosive properties of the composite coatings with different composition of PNVPY/CB/PVB are discussed.

## 2. Experimental

### 2.1. Materials

Pure zinc sheets with a thickness of 1 mm were supplied by China New Metal Materials Technology Co., Ltd. (Zhangjiagang, China). The zinc sheets were cut into small pieces with an exposed surface of 20 × 10 mm^2^ using wire electrical discharge machining for corrosion experiments. The surface of the zinc was polished with 600, 800 and 1000 grit SiC paper, respectively. Then, they were immersed in ethanol using an ultrasonic bath for 20 min and dried in a nitrogen stream. Conductive CB (super C65) and PVB were purchased from Timcal Company (Shanghai, China) and Sinopharm Chemical Reagent Co. Ltd. (Beijing, China), respectively. All the solvents were obtained from Tianli Chemical Reagent Co. (Tianjin, China) without any pretreatment. A MODEL KW-4A Spin Coater (Siyouyen Ltd., Beijing, China) was used for distributing the DMF solution of PNVPY/CB/PVB composites evenly on zinc substrate. The electrochemistry analysis was recorded by a CHI760 electrochemical workstation (CH instruments Ltd., Shanghai, China). 

### 2.2. The Synthesis of PNVPY Nanoparticles

Well-dispersed sulfate doped PNVPY nanoparticles for the coating uses were prepared via UV-catalytic polymerization with H_2_O_2_ as an oxidant and polyvinyl pyrrolidone (PVP) as a stabilizer, referred to as the green synthesis method developed by our group [23]. The synthesis process was shown as follows: A certain amount of *N*-(vinyl)pyrrole (NVPY) was added to 30 mL PVP aqueous solution. After magnetic stirring (800 rpm) for 10 min, an additional 30 mL aqueous solution mixture of H_2_O_2_ and H_2_SO_4_ was introduced to the above solution. Under UV irradiation (253.7 nm), the polymerization reaction was initiated and proceeded for 5 h at room temperature. The PNVPY nanoparticles were obtained at a centrifugal speed of 12,000 rpm for 10 min.

### 2.3. The Preparation of Composite Coating and Coated Zinc

The preparation process of anti-corrosion coatings is shown as follows: PNVPY nanoparticles with different concentrations (1.9 wt.%, 6.4 wt.% and 8.9 wt.%) were added in 1.6 mL *N*,*N*-dimethylformamide (DMF) and stirred thoroughly by magnetic stirrer for 30 min at room temperature. Later on, CB particles were dispersed in the above solution and the mixture was treated with ultrasonic for 10 min. At last, a certain amount of PVB was added in and stirred for 4 h at 35 °C. Then, the composite dispersions were spin-coated directly on zinc with a speed of 2500 rpm and dried at 60 °C for 2 h. The spin time was adjusted to maintain the same thickness of the coating of different samples. The dispersion of 13.9 wt.% PVB dissolved in 1.6 mL DMF was spin-coated once again at the same spin speed on the composite films and dried at 60 °C for 2 h. The thickness of the coatings here was maintained at about 7 μm according to the similar system investigated by Bai et al. [21] to compare the anti-corrosion properties of the coatings. The concentrations of composite coatings with different ratio of PNVPY/CB/PVB are listed in Table 1.

### 2.4. Characterization Methods

The morphology of PNVPY was observed by dropping the diluted reaction solution of PNVPY directly onto the silicon wafer using a JSM 7000M scanning electron microscope (SEM) (JEOL Ltd., Tokyo, Japan) as soon as the polymerization of NVPY finished. The particle size and size distributions of PNVPY particles were analyzed by the dynamic lighting scattering (DLS) technique using a Nano-ZS90 Malvern instrument (Malvern Instruments Ltd., Worcestershire, UK). The tests were performed by dispersing the PNVPY powders into several solvents. The morphologies of PNVPY powders dissolved in DMF were observed by a JEM-2100 transmission electron microscope (TEM) (JEOL Ltd., Tokyo, Japan ). The CB nanoparticles were added to ethanol with ultrasonic treatment, then dropped onto the silicon wafer for SEM (JSM 7000M) observation. The PNVPY/CB/PVB composite coating film exfoliated from the coated zinc was quenched in liquid nitrogen. Both the upper surface and cross section were observed (JSM 7000M).

The electrochemistry analysis was performed in a single compartment three electrodes cell (bare zinc and coated zinc as the working electrode with an exposed area of 2 cm^2^, a platinum plate as the counter electrode and a saturated calomel electrode as the reference electrode) at room temperature in a 3.5% NaCl solution. The open circuit potential (Eocp) measurements were performed for 400 s after the samples were immersed in 3.5% NaCl solution for 30 min. Electrochemical Impedance Spectroscopy (EIS) data was recorded in the frequency ranging from 100 kHz to 100 mHz by immersing the electrodes into the 3.5% NaCl solution. The potentiodynamic polarization curves were obtained starting from the open circuit potential (OCP) with a scan rate of 1 mV s^−1^ and varying the potential to 300 mV in a set of experiments (anodic region of the Tafel plot) and to 300 mV in another set of experiments (cathodic region of the Tafel plot). Oxygen was not removed from the NaCl solution before these experiments.

## 3. Results and Discussion

### 3.1. Characterization of PNVPY Nanoparticles

**Figure 1:**
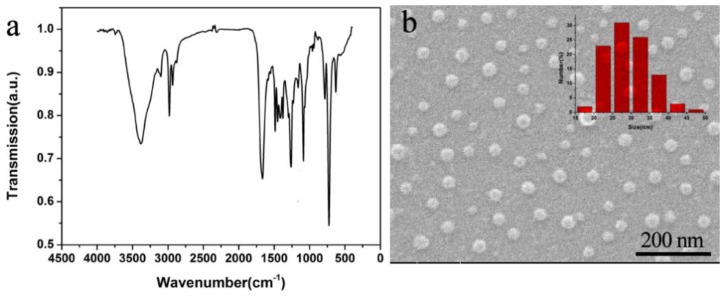
(**a**) Fourier Transform infrared spectroscopy and (**b**) morphologies of the PNVPY nanoparticle.

The structure characteristic and the morphology of sulfate doped PNVPY were investigated as shown in Figure 1. It can be seen from Figure 1a that the absorption peaks at 1551 and 1487 cm^−1^ correspond to the aromatic ring in PNVPY. The absorptions at 1660 and 3099 cm^−1^ represent the double bond. The peak that appeared at 781 cm^−1^ is attributed to the a-substituted five-membered heterocyclic ring compound [24]. The absorption bands at 1375 and 1087 cm^−1^ correspond to asymmetric and symmetric S(=O)_2_ stretching [25,26]. The morphology of PNVPY was observed by dropping the diluted reaction solution of PNVPY directly onto the silicon wafer as soon as the polymerization of NVPY finished. It can be seen in Figure 1b that PNVPY nanoparticles are spherical with diameters ranging from 22 nm to 58 nm, and the average size is around 38 nm.

### 3.2. Morphology Analysis of Coatings

**Figure 2:**
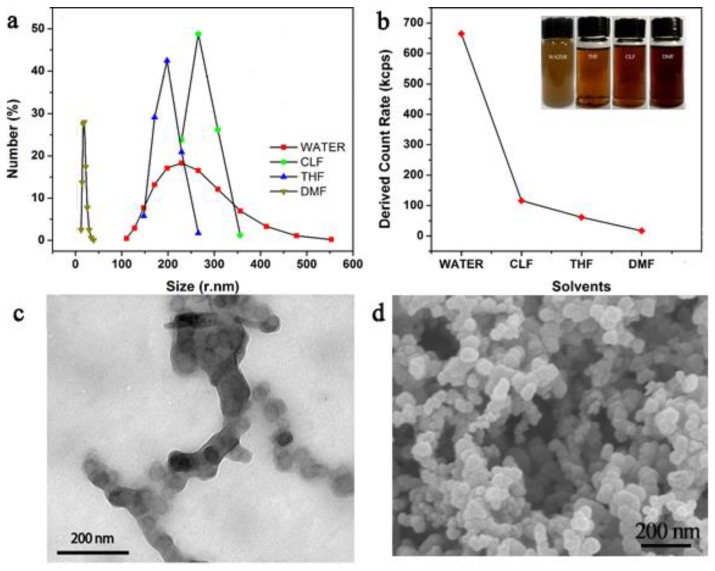
(**a**) The size distribution of PNVPY particles in different solvents. (**b**) The value of Derived Count Rate of PNVPY particles dispersed in different solvents (solution concentration = 0.81 g/L). The inset is the photos of PNVPY particles dispersed in different solvents. (**c**) Transmission electron microscope (TEM) photograph of PNVPY particles obtained from its *N*,*N*-dimethylformamide (DMF) dispersion. (**d**) Scanning electron microscope (SEM) photograph of CB particles obtained from its ethanol dispersion.

The poor dispersibility of conductive polymers in versatile organic solvents restrains their applications on spin-coating. In our study, PNVPY powders can be dispersed well in water, tetrahydrofuran (THF), chloroform (CLF), and *N*,*N*-dimethylformamide (DMF) (inset of Figure 2b). The size distribution of PNVPY powders in several solvents is measured by DLS, as shown in Figure 2a. It was found that the size of PNVPY particles decreases gradually from about 300 nm to 1 nm in H_2_O, CLF, THF and DMF, respectively. It can be attributed to the aggregation of PNVPY particles, especially in H_2_O, CLF, THF. This phenomenon also can be verified by the decrease in the value of Derived Count Rate in H_2_O, CLF, THF and DMF, respectively, as shown in Figure 2b. Moreover, the average size of particles in DMF is about 30 nm, which is in accordance with its TEM photograph (the spherical PNVPY particle with an average size of about 30 nm), as shown in Figure 2c. Therefore, DMF is a favorable solvent to disperse PNVPY particles for the preparation of the composite coatings. The SEM micrograph of CB is shown in Figure 2d. It is clear that the CB particles are grouped into chains, forming spatial network channel-like grape clusters. The mesh chains are stacked tightly, leading to a large specific surface area and high load of CB particles per unit of mass, which facilitated the formation of the chain conduction structure in the polymer.

**Figure 3:**
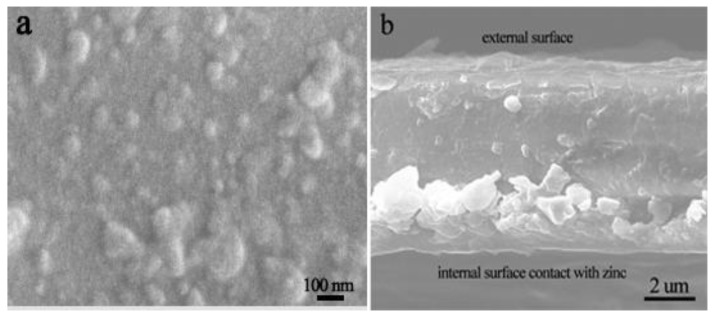
(**a**) Surface and (**b**) cross-section photos of the PNVPY/CB/PVP film exfoliated from coated zinc.

The morphologies of the upper surface and cross section of the PNVPY/CB/PVB coating exfoliation from the coated zinc (Sample 4) are shown in Figure 3. The film exhibits a relatively flat surface with many granules and rare aggregation of PNVPY and CB as observed from Figure 3a. As blended with PNVPY, part of the CB nanoparticles may act as a bridge to ensure the electronic connection between the PNVPY nanoparticles in the coating. Meanwhile, as can be seen in Figure 3b, there are more nanoparticles near the internal surface contact with zinc.

### 3.3. Corrosion Studies by Electrochemical Method

#### 3.3.1. Open Circuit Potential (OCP) Measurement

**Figure 4:**
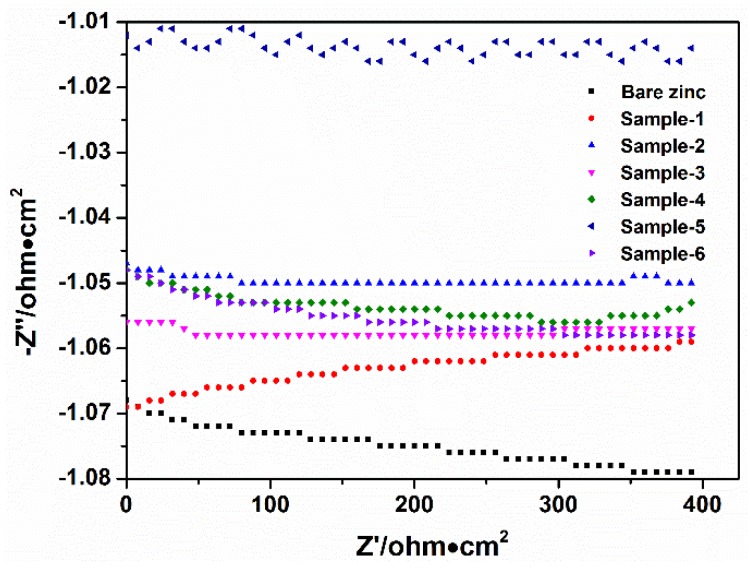
Open circuit potential (OCP) variation with time for samples immersed in a 3.5% NaCl solution at room temperature (25 °C).

Figure 4 illustrates the variation of OCP at different times for coated and uncoated zinc immersed in a 3.5% NaCl solution. The trends of OCP variation with time of bare zinc exhibit a gentle shift of potential towards the cathodic direction. The OCP of the bare zinc is about −1079 mV vs. SCE (Saturated calomel electrode) at the end of the immersion time. The OCP of pure PVB coating (Sample 1) shifts gradually towards the anodic direction and maintains at around −1059 mV (SCE). Moreover, the PVB/CB composite coating (Sample 3) maintains a small fluctuation at about −1057 mV. In contrast, the OCP levels of the zinc with PVB/CB/PNVPY composite coating of dosage 1.9% (Sample 4), 6.4% (Sample 5) and 8.9% (Sample 6) are higher than bare zinc and Sample-3. Especially, the OCP level of Sample 5 is as high as about −1010 mV, indicating it has a potentially favorable anti-corrosion effect on zinc.

#### 3.3.2. Electrochemical Impedance Spectroscopy

**Figure 5:**
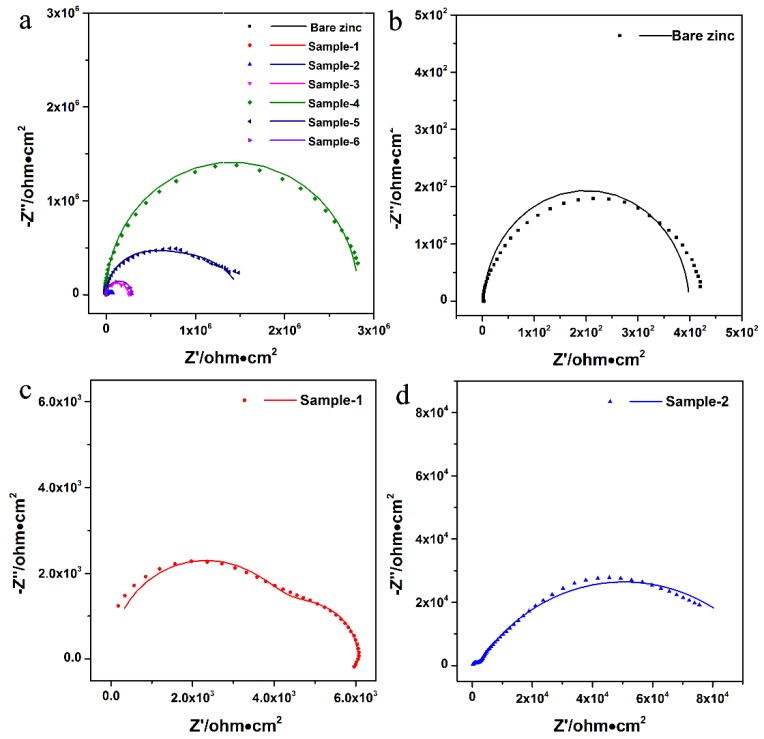
(**a**–**d**) Nyquist plots of electrochemical impedance spectroscopy (EIS) (dots) and fits of a ZsimpWin (version 3.60, lines) operated immersed in a 3.5% NaCl solution at room temperature (25 °C). The R^2^ values from EIS data of bare zinc, Sample 1, Sample 2, Sample 3, Sample 4, Sample 5 and Sample 6 were 0.9625, 0.9376, 0.9967, 0.9025, 0,9499 and 0.9448, respectively.

Electrochemical impedance spectroscopy (EIS) is a useful and non-destructive method in studying the corrosion mechanism. Figure 5 shows the EIS of all samples. The Nyquist plots obtained from bare zinc and coated zinc in the condition of open circuit potential are displayed in Figure 5a. The Nyquist plot of bare zinc (Figure 5b) shows a small arc with the lowest impedance value as compared to other specimens. It can be seen in Figure 5c that the specimen of pure PVB coated zinc has a larger impedance value than bare zinc. The impedance value of Sample 2 increased by an order of magnitude due to the addition of PNVPY (1.9 wt.%) to the PVB coating (Figure 5d). It is considered that the Fermi-level misalignment between zinc and the conductive polymer may cause an improved corrosion protection on zinc only by a passive and not by an active function [4]. Moreover, an obvious high impedance value of the zinc coated with PNVPY/CB/PVB composite coating appears as shown in Figure 5a (Sample 4, Sample 5 and Sample 6). It indicates that the addition of CB matches Fermi resonance between conducting polymers and the surface of the zinc. Interestingly, the impedance value of the specimen does not increase with the increase in PNVPY dosage from 6.4 wt.% to 8.9 wt.%. There is a significant reduction of the radius of the semicircle when the PNVPY content increases to 6.4 wt.% and 8.9 wt.%. It is inferred that the proportion of the CB is reduced in the composite when the PNVPY content increases, which cannot solve the problem of electronical decoupling effectively.

**Figure 6:**
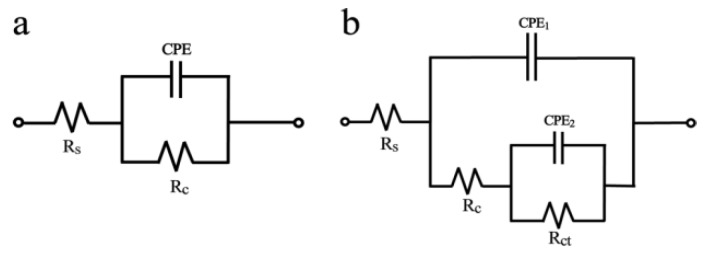
Equivalent circuit model of (**a**) uncoated zinc and (**b**) coated zinc. CPE_1_ and CPE_2_ are coating film capacitance and its double-layer capacitance in 3.5% NaCl solution, respectively. R_s_, R_c_ and R_ct_ are solution resistance, coating resistance and charge transfer resistance.

The equivalent circuits are shown in Figure 6. The corresponding electrochemical parameters extracted by fitting EIS plots with the Zsimpwin program are summarized in Table 2. CPE is a constant phase element, and CPE_1_ and CPE_2_ are used in place of coating capacitance and double-layer capacitance in order to give more accurate fits to the experimental results. R_c_ is attributed to the pore resistance and signifies the performance of the surface coating [15,27], which is inversely proportional to the defects (pores) in a film. Its value can be taken as a measure of the porosity and the degree of degradation of a coating film.

The R_c_ values for different samples are shown in Table 2. A low R_c_ (395 Ω cm^2^) for bare zinc indicates the presence of a porous layer of corrosion product on its surface. For this reason, chloride ions continually diffuse to the metal surface, causing a decreased R_c_. The measured R_c_ of zinc coated with pure PVB coating is almost one order of magnitude higher than the uncoated zinc because of the physical barrier effect. The R_c_ increases more than one order of magnitude for Sample 3 as compared to bare zinc. Sample 4 shows the highest R_c_ (2.71 × 10^6^) among the specimens tested, indicating its superior barrier property. Moreover, the value of R_c_ decreases when the content of PNVPY increases from 6.4 wt.% to 8.9 wt.% in the composite coatings.

#### 3.3.3. Potentiodynamic Polarization

**Figure 7:**
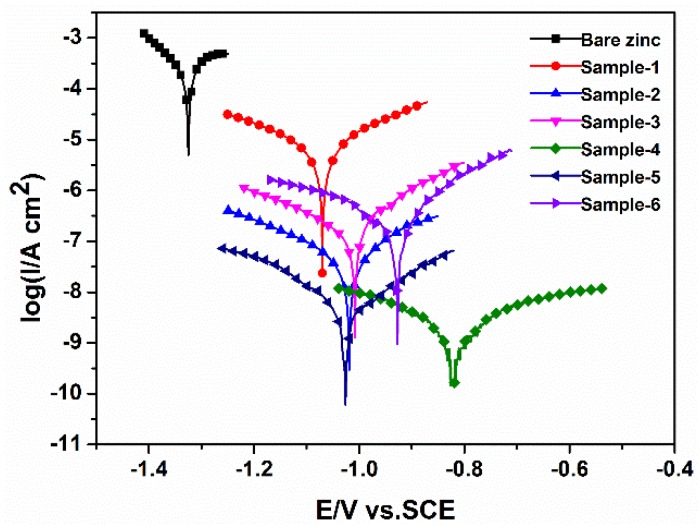
Potentiodynamic polarization curves of samples immersed in a 3.5% NaCl solution at room temperature (25 °C).

The potentiodynamic polarization method was also employed in order to compare the corrosion prevention ability of various polymer coatings on Zn [28]. A higher corrosion potential (E_corr_) and a lower corrosion current (i_corr_) is advantageous to effective anti-corrosion coating. Figure 7 shows the potentiodynamic polarization curves of samples immersed in a 3.5% NaCl solution at room temperature (25 °C). Meanwhile, to reveal the effect of conductive filler on the anti-corrosion behavior of the coating in detail, the electrochemical parameters obtained by Tafel extrapolation of the curves in Figure 7 are listed in Table 3. As can be seen from Figure 7 and Table 3, the corrosion current density (i_corr_) of Sample 1 (i_corr_ = 8.278 × 10^−6^ A/cm^2^) is almost three orders of magnitude less than the bare zinc. It is reasonable that polymer coatings have good resistance towards diffusive ions [29]. When the carbon black is added as the inorganic filler to the PVB coating (i_corr_ = 2.231 × 10^−7^ A/cm^2^), it acts as a mechanical integrity significantly improving the physical barrier ability against the penetration of aggressive chloride ions, which is the same function as other conventional coatings/paints that inhibit the penetration of ions, protecting the metal surface [30,31,32]. Meanwhile, the addition of conductive CB avoids forming an insulating layer that will cause electrical decoupling (Fermi-level misalignment) at the PNVPY/metal interface and has a seemingly low potential (Sample 3) [21]. The i_corr_ of the coated zinc further decreased to 1.245 × 10^−9^ A/cm^2^, 5.349 × 10^−9^ A/cm^2^ and 1.924 × 10^−7^ A/cm^2^, when the zinc was spin-coated with the composite coatings of PNVPY/CB/PVB containing PNVPY nanoparticles of 1.9 wt.%, 6.4 wt.% and 8.9 wt.%, respectively. In particular, Sample 4 has extremely high E_corr_ (−0.821 V) and low i_corr_ (5.349 × 10^−9^ A/cm^2^), showing excellent corrosion protection efficiency. In addition, the corrosion current increases with the increase of PNVPY content, which demonstrates excessive conducting polymers added in coatings are not good for anti-corrosion. The corrosion protection efficiency (% P.E.) is defined by the measured i_corr_ {corrosion current density of uncoated Zn (i^0^_corr_) and the corrosion current density of coated Zn (i^c^_corr_)} values in the Equation (1) [20].

% P.E. = (i^0^_corr_ − i^c^_corr_)/i^0^_corr_ × 100 (1)

The highest calculated % P.E. of Sample 4 (99.99%) shows outstanding corrosion resistance, which is coincident with the result of lowest corrosion current. The results were better than those reported in other literature [2,33,34,35].

### 3.4. Immersion Test of the Coatings without Defect

**Figure 8:**
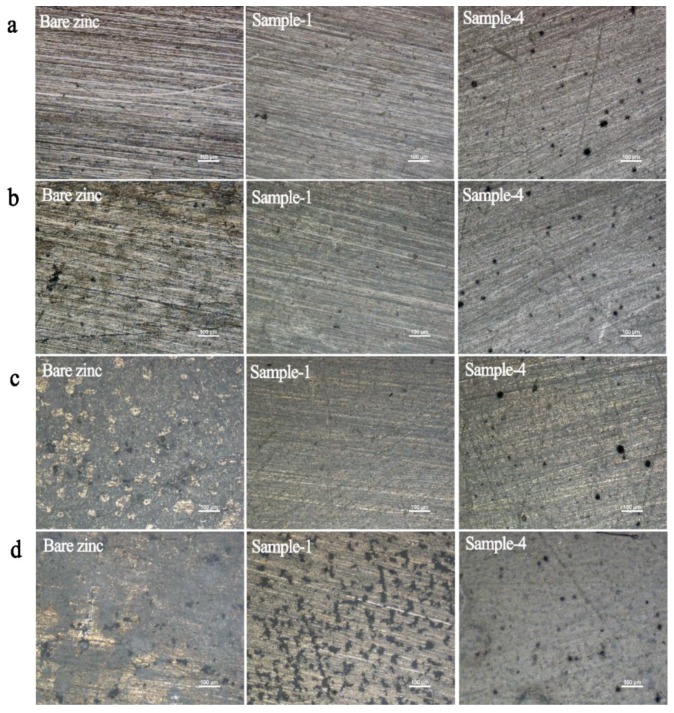
Photograph of bare zinc, PVB coated zinc (Sample 1) and PVB blended with 1.9 wt.% PNVPY and 2.3 wt.% CB coated zinc (Sample 4) in different immersion times (**a**) 0 h (**b**) 24 h (**c**) 48 h (**d**) 168 h. The specimens were tested in 3.5% NaCl solution at room temperature (25 °C).

Three samples were chosen to assess corrosion protection performance for a long time by immersing them in 3.5% NaCl solution, including bare zinc, pure PVB coated zinc (Sample 1) and PVB blended with 1.9 wt.% PNVPY and 2.3 wt.% CB coated zinc (Sample 4). Figure 8 clearly shows that uncoated zinc suffers a severe corrosion, while the Sample 1 nearly has no significant change after 48 h. However, a serious corrosion appears on the surface after 168 h, as a consequence of the penetration of corrosive ion into the metal surface, damaging the physical barrier. Sample 4 shows no significant change after 168 h in 3.5% NaCl, indicating its remarkably high corrosion protection. Consequently, Sample 4 has optimum performance against corrosive in seawater conditions.

### 3.5. Anti-Corrosion Mechanism Discussion

Compared to the coating with pure PVB, PNVPY/CB/PVB composite coatings exhibit superior anti-corrosion properties in both physical barrier and chemistry protection as investigated above. It is inferred that organic coatings possess the ability to prevent ion penetration. The PNVPY and CB nanoparticles act as a physical barrier against the chloride ion and oxygen penetrating to the metal surface. Anodic protection by electronic barrier effect is one of the most important factors for metal anticorrosion.

**Figure 9:**
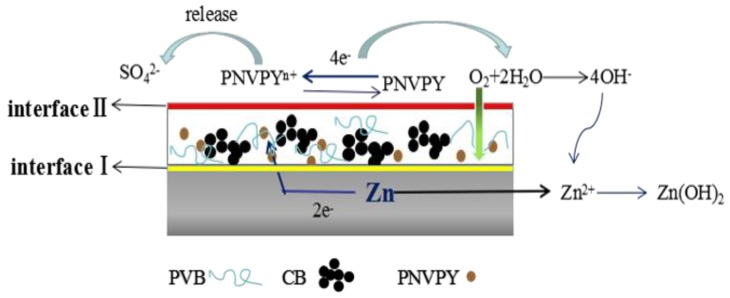
The illustration of the corrosion mechanism of the PNVPY/CB/PVB composite coating.

Based on the morphology analysis of the cross section of the PNVPY/CB/PVB coating exfoliation from the coated zinc (Sample 4) as shown in Figure 3, the schematic diagram of the corrosion mechanism is drafted in Figure 9. CPs have strong oxidation potential on the zinc surface. As a result, well-dispersed sulfate doped PNVPY will release doped anions and accept electrons when corrosion occurred, intercepting electron transport between metal surface and electrolyte [36]. CB acts as a bridge between the PNVPY nanoparticles and zinc to ensure the electronic contact and transfer. Besides, PNVPY shifts the reaction site of oxygen reduction from metal/polymer interface (interface I) into the polymer/electrolyte interface (interface II) [37,38]. At the moment, the concentration of oxygen and water is gradually decreasing from interface II to interface I. Therefore, the corrosion rate of the zinc surface is slowed down to achieve the purpose of protection.

## 4. Conclusions

In conclusion, to achieve high corrosion resistance of zinc, an environment friendly and easy produced PNVPY/CB/PVB composite coating is obtained. The anti-corrosion properties of the coatings with different content of conductive filler were evaluated using OCP, EIS, and potentiodynamic polarization techniques by immersing them into a 3.5% NaCl solution at 25 °C. The electrochemical measurements revealed that the PVB coating with 1.9 wt.% PNVPY and 2.3 wt.% CB significantly improves the corrosion resistance properties of zinc and shows a maximum corrosion protection efficiency up to 99.99%. The immersion results reveal that this coating restrains the spread of corrosion and forms a superior anti-corrosive coating for zinc, while both bare zinc and PVB coated zinc, however, show a severe corrosion for 148 h in 3.5% NaCl solution.

## Figures and Tables

**Table 1:** The concentration of composite coatings with different ratios of PNVPY/CB/PVB.

Sample	PNVPY (wt.%)	CB (wt.%)	PVB (wt.%)
1	0	0	100
2	2	0	98
3	0	2.3	97.7
4	1.9	2.3	95.8
5	6.4	2.2	91.4
6	8.9	2.1	89

**Table 2:** Impedance data for bare zinc and coated zinc with the composite coatings in 3.5% NaCl.

Sample	CPE_1_	n_1_	R_c_ (Ω cm^2^)	CPE_2_	n_2_	R_ct_ (Ω cm^2^)
Bare zinc	1.48 × 10^5^ ± 5.8%	0.98 ± 2.0%	395 ± 5.4%	-	-	-
1	1.51 × 10^9^ ± 1.6%	0.95 ± 2.0%	4.59 × 10^3^ ± 2.9%	5.21 × 10^8^ ± 1.7%	0.98 ± 0.8%	1.48 × 10^3^ ± 3.5%
2	1.54 × 10^8^ ± 4.0%	0.88 ± 5.2%	3.89 × 10^3^ ± 2.8%	2.24 × 10^6^ ± 2.9%	0.63 ± 3.3%	8.94 × 10^4^ ± 4.4%
3	4.24 × 10^10^ ± 3.6%	0.96 ± 4.2%	2.16 × 10^5^ ± 5.5%	3.08 × 10^6^ ± 2.1%	0.66 ± 5.8%	2.86 × 10^4^ ± 3.9%
4	3.55 × 10^10^ ± 2.3%	0.95 ± 3.5%	2.71 × 10^6^ ± 2.0%	1.57 × 10^7^ ± 1.8%	0.78 ± 4.6%	2.12 × 10^5^ ± 2.7%
5	1.67 × 10^10^ ± 2.2%	0.94 ± 3.6%	1.65 × 10^6^ ± 1.9%	7.27 × 10^9^ ± 4.0%	0.76 ± 2.5%	1.42 × 10^5^ ± 3.1%
6	3.61 × 10^10^ ± 5.0%	0.92 ± 3.4%	2.65 × 10^5^ ± 6.1%	6.26 × 10^6^ ± 2.5%	0.82 ± 4.3%	2.60 × 10^4^ ± 2.7%

**Table 3:** Electrochemical parameters obtained by Tafel extrapolation in 3.5% NaCl solution.

Sample	E_corr_ (V)	i_corr_ (A/cm^2^)	% P.E.
Bare zinc	−1.320 ± 4.3%	2.665 × 10^−4^ ± 3.6%	-----------
1	−1.069 ± 3.5%	8.278 × 10^−6^ ± 6.1%	96.89
2	−1.015 ± 3.8%	4.868 × 10^−8^ ± 6.1%	99.98
3	−1.009 ± 6.6%	2.231 × 10^−7^ ± 3.7%	99.92
4	−0.821 ± 5.0%	1.245 × 10^−9^ ± 5.2%	99.99
5	−1.030 ± 7.6%	5.349 × 10^−9^ ± 6.3%	99.98
6	−0.930 ± 4.9%	1.924 × 10^−7^ ± 4.0%	99.93
